# Trends and disparities in gastrointestinal hemorrhage-related mortality in individuals with diabetes in the United States from 1999 to 2023: A cross-sectional study

**DOI:** 10.1097/MD.0000000000048293

**Published:** 2026-04-17

**Authors:** Moiz Ul Haq Hashmi, Ramisha Chaudhary, Sayed Jawad Hussaini, Krish Patel, Ghulam Mustafa Ali Malik, Ramish Hannat, Muddassir Khalid, Muhammad Riyyan, F.N.U. Farukhuddin, Muhammad Saeed Qazi, Laiba Akhlaq

**Affiliations:** a Department of Medicine, Nishtar Medical University, Multan, Punjab, Pakistan; b Department of Medicine, Dow Medical College, Karachi, Sindh, Pakistan; c Department of Medicine, C. U. Shah Medical College, Surendranagar, Gujarat, India; d Department of Medicine, Services Institute of Medical Sciences, Lahore, Punjab, Pakistan; e Department of Medicine, Brown University, Providence, RI; f Department of Internal Medicine, Brown University, Providence, RI; g Department of Medicine, Liaquat University of Medical and Health Sciences, Jamshoro, Sindh, Pakistan.

**Keywords:** CDC WONDER, diabetes, disparities, gastrointestinal hemorrhage, mortality trends, United States

## Abstract

Gastrointestinal hemorrhage in patients with diabetes is associated with a higher risk of death. This study examined the trends and disparities in U.S. adult mortality due to coexisting diabetes and gastrointestinal hemorrhage. We analyzed CDC WONDER data (1999–2023) for adults (≥25 years), where gastrointestinal hemorrhage and diabetes were listed together as underlying or contributory causes of death. Age-adjusted mortality rates (AAMRs) per 100,000 U.S. population were calculated, and trends in AAMRs were analyzed using Joinpoint regression software. Relevant sensitivity analyses were conducted to verify the direction of the overall trend. Sociodemographic and geographical disparities in relevant trends were also explored. From 1999 to 2023, gastrointestinal hemorrhage and diabetes mellitus concurrently led to 80,833 deaths among U.S. adults, at an AAMR of 1.45/100,000. There was no net change in mortality rates over this period (AAPC −0.16; 95% CI: −0.58–0.15). In contrast, the AAMRs from gastrointestinal hemorrhage without fatal diabetes decreased (AAPC −1.03; 95% CI: −1.56 to −0.52). Males exhibited higher mortality rates from coexisting gastrointestinal hemorrhage and diabetes (Males 1.84 vs Females 1.15). Non-Hispanic American Indian/Alaska Native (2.97) and NH Black/African American (2.32) populations had the highest AAMRs. Disproportionately high overall rates were observed in the West (1.53) and South (1.48) regions. Moreover, AAMRs were notably higher in rural areas (rural: 1.72 vs urban: 1.31). Further evidence is required to establish the bidirectional mortality risks associated with gastrointestinal hemorrhage in patients with diabetes. Nonetheless, the disproportionate and persistent mortality burden in the U.S. demands attention.

## 1. Introduction

Gastrointestinal hemorrhage is one of the most important causes of hospital admission in the United States.^[1]^ It is broadly classified into upper and lower gastrointestinal bleeding, and often arises as a severe consequence of gastrointestinal disorders.^[2]^ Despite advances in pharmacological and surgical treatments, the overall burden of gastrointestinal hemorrhage in the United States remains high. According to CDC WONDER, it led to 803,987 deaths among the adult U.S. population between 1999 and 2020.^[3]^

This burden is further compounded by comorbid conditions, particularly metabolic and cardiovascular disorders. Among these, diabetes mellitus remains one of the most common and consequential. In 2023, diabetes was solely responsible for approximately 95,190 deaths in the U.S. and affected almost 15.8% of the adult population.^[4]^ Beyond its independent burden, diabetes frequently coexists with gastrointestinal hemorrhage and is linked to poorer outcomes.^[5,6]^ According to an observational study, 11.5% of patients hospitalized with diabetic ketoacidosis developed gastrointestinal hemorrhage, and these individuals had higher odds of in-hospital mortality compared with those without hemorrhage.^[7]^

Poor glycemic control, reported in more than 50% of patients with diabetes,^[8]^ predisposes them to acid-related upper gastrointestinal disorders and mucosal injury, and thus higher odds of gastrointestinal hemorrhage.^[9]^ Diabetic nephropathy, resulting from chronic kidney damage due to diabetes, has also been linked to variceal bleeding even in the absence of liver damage.^[10]^ Furthermore, diabetes-related autonomic neuropathy and vascular dysfunction have been known to impair physiological responses to bleeding, raising mortality risk associated with hemorrhage.^[11]^ It is also essential to consider that many patients with diabetes receive concomitant antiplatelet and anticoagulant treatments for cardiovascular comorbidities, further increasing the risk of bleeding.^[12]^ Taken together, these factors complicate the management of gastrointestinal hemorrhage in diabetes, often necessitating closer hemodynamic monitoring, stricter glycemic control, and more cautious adjustment of medications than in the general population. Due to these well-documented risks and the need for specifically tailored management of gastrointestinal bleeding among patients with diabetes, understanding the actual mortality burden becomes particularly important.

Despite this, population-level data on the mortality burden of co-existent gastrointestinal hemorrhage and diabetes mellitus in the U.S. remain scarce. We therefore conducted this analysis to study the national mortality burden due to co-contributing gastrointestinal hemorrhage and diabetes mellitus and to highlight how this burden differed among various sociodemographic and geographic subgroups between 1999 and 2023.

## 2. Methodology

This study conforms to the Strengthening the Reporting of Observational Studies in Epidemiology (STROBE) statement.

### 2.1. Study setting and population

This study is a retrospective analysis of gastrointestinal bleeding and diabetes-related mortality in the United States from 1999 to 2023. The data were obtained from the Centers for Disease Control and Prevention Wide-Ranging Online Database for Epidemiologic Research (CDC WONDER).^[13]^ This database was used to identify records with diabetes mellitus and gastrointestinal bleeding listed as underlying or contributing causes of death. The International Classification of Diseases, 10th revision codes (ICD-10 codes) used for both of these conditions are listed in the “Supplementary Data File (Supplemental Digital Content, https://links.lww.com/MD/R641). The CDC WONDER database only contains anonymized, publicly available data; therefore, the study was exempt from Institutional Review Board approval. The study adhered to the Strengthening the Reporting of Observational Studies in Epidemiology (STROBE) guidelines.^[14]^

### 2.2. Data extraction

We extracted annual mortality data, spanning the years 1999 to 2023. Within that data extracted, further classifications, including demographic and regional groups, were collected. This stratification included sex, race/ethnicity, urban-rural classification, census region, and states. In our analysis, racial/ethnic groups were defined as non-Hispanic (NH) White, NH Black/African American, NH American Indian/Alaskan Native, NH Asian/Pacific Islander, and Hispanic or Latino people. Data were divided into rural and urban classifications according to the National Center for Health Statistics Urban-Rural Classification Scheme, which divided data into urban/metropolitan areas (large metropolitan area [population ≥ 1 million], medium/small metropolitan area [population 50,000–999,999]) and rural/non-metropolitan (population < 50,000) areas as per the 2013 United States census classification.^[15]^ The Census Bureau definitions were used to divide data into the regional classifications of Northeast, Midwest, South, and West.^[16]^

### 2.3. Statistical analysis

Gastrointestinal hemorrhage and diabetes-related age-adjusted mortality rates were extracted. We standardized the age-adjusted mortality rate (AAMR) per 100,000 persons using the 2000 United States standard population as previously described.^[17]^ To determine trends in mortality within the study period, we used the Joinpoint Regression Program (Joinpoint version 5.4.0, available from the National Cancer Institute, Bethesda).^[18]^ The annual percentage change (APC) and average annual percentage change (AAPC) with 95% confidence intervals for the respective AAMRs were calculated using the Monte Carlo permutation test. A Bonferroni-type correction was applied to control for multiple testing across the Joinpoint models. The change in rates was considered significant if the slope describing the change was significantly different from zero based on a 2-tailed *t*-test (*P* < .05 was considered statistically significant). Joinpoint regression assumes that temporal trends can be modeled as a series of contiguous log-linear segments, with constant annual percentage change within each segment. The method further assumes independence and homoscedasticity of residuals and a Poisson distribution of mortality counts. All rates were log-transformed to stabilize variance, consistent with standard Joinpoint methodology.

For sensitivity analysis of the overall trend, we restricted the analysis to deaths where gastrointestinal hemorrhage was the underlying cause and diabetes mellitus was listed as a contributory cause. We further conducted pairwise comparisons among related cohorts using the test of parallelism in the Joinpoint regression software.

## 3. Results

### 3.1. Overall mortality trends due to gastrointestinal hemorrhage and diabetes

**Figure 1.**
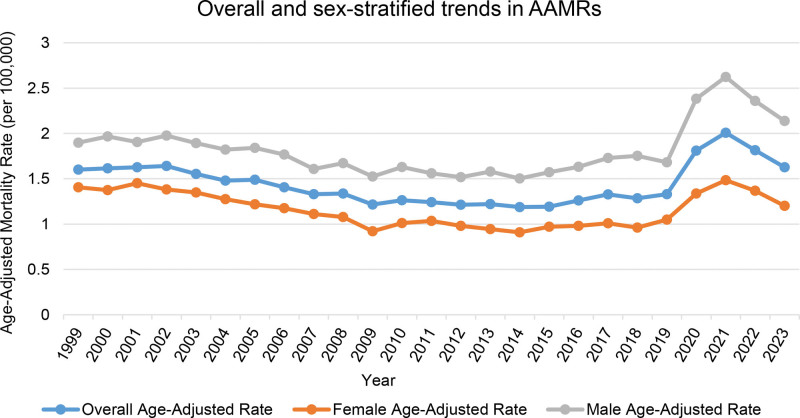
Overall and sex-specific mortality trends from coexisting diabetes and gastrointestinal bleeding, 1999–2023; Age-adjusted mortality rates (AAMRs) per 100,000 U.S. adults aged ≥ 25 yr, overall and stratified by sex. AAMR = age-adjusted mortality rate.

In epidemiologic terms, an absolute AAPC between −0.5% and +0.5% was interpreted as relative stability in mortality rates, whereas values exceeding ±1.0% were considered indicative of clinically meaningful temporal change. Between 1999 and 2023, gastrointestinal hemorrhage and diabetes concurrently contributed to 80,833 deaths among U.S. adults aged ≥ 25 years. The combined AAMR over this period was 1.45 per 100,000 (AAPC −0.16; 95% CI: −0.58–0.15). AAMR declined from 1.6 in 1999 to 1.21 in 2012 (APC −2.62*; 95% CI: −4.55–−2.02), then remained statistically unchanged through 2018. AAMR increased sharply between 2018 (1.29) and 2021 (2.01) (APC 16.79*; 95% CI: 12.25–20.05), before falling to 1.63 in 2023 (APC −10.25*; 95% CI: −14.94–−5.69) (Fig. 1, Table 1, Table 2, Table S1, Supplemental Digital Content, https://links.lww.com/MD/R640).

**Table 1:** Raw deaths, population, and combined age-adjusted mortality rates (per 100,000; standardized to the 2000 US standard population) due to coexistent gastrointestinal hemorrhage and diabetes among the adult U.S. population. Data is stratified by demographic and geographical subgroups and ranges from 1999 to 2023.

	Total deaths	Total population	Overall age-adjusted mortality rates per 100,000 U.S. adult population (95% CI)
Overall			
	80,833	5,150,005,971	1.45 (1.44–1.46)
Sex			
Female	37,218	2,665,276,200	1.15 (1.14–1.16)
Male	43,615	2,484,729,771	1.84 (1.82–1.85)
Race/Ethnicity			
Hispanic	8134	702,028,728	1.83 (1.79–1.87)
NH American Indian/Alaska Native*	974	27,233,387	2.97 (2.78–3.16)
NH Asian/Pacific Islander	3129	281,715,534	1.45 (1.40–1.50)
NH Black/African American	11,820	602,204,655	2.32 (2.28–2.36)
NH White	56,776	3,528,912,956	1.30 (1.28–1.31)
Census regions			
Northeast	14,209	946,230,388	1.30 (1.28–1.32)
Midwest	17,902	1,108,988,111	1.43 (1.41–1.46)
South	30,365	1,912,217,015	1.48 (1.47–1.50)
West	18,357	1,182,570,457	1.53 (1.50–1.55)
Urbanization†			
Urban	51,957	3,795,213,822	1.31 (1.30–1.32)
Rural	14,277	678,634,169	1.72 (1.69–1.75)

* Age-adjusted mortality rates (AAMRs) for non-Hispanic American Indians or Alaska Natives were unavailable for multiple years between 1999 and 2006 (due to CDC dada restrictions), and therefore could not be analyzed. Consequently, the total AAMRs for this group were calculated from 2007 onwards.

† Urbanization level data is limited from 1999–2020.

**Table 2:** Annual percentage changes and average annual percentage changes in gastrointestinal hemorrhage and diabetes mellitus-related age-adjusted mortality rates among U.S. adults from 1999–2023.

Variable	Lower endpoint	Upper endpoint	APC (95% CI)	*P*-value for APC	AAPC (95% CI)	*P*-value for AAPC
**Overall**						
	1999	2012	−2.62* (−4.55–−2.02)	.007598	−0.16 (−0.58–0.15)	.307938
	2012	2018	0.98 (−1.64–4.41)	.411118		
	2018	2021	16.79* (12.25–20.05)	<.000001		
	2021	2023	−10.25* (−14.94–−5.69)	<.000001		
**Sex**						
**Females**	1999	2002	0.43 (−2.66–6.03)	.832633	−0.60* (−0.97–−0.23)	<.000001
	2002	2009	−4.96* (−8.31–−3.79)	.018796		
	2009	2018	−0.41 (1.60–−0.90)	.451110		
	2018	2021	16.25* (12.71–18.81)	<.000001		
	2021	2023	−10.25* (−14.06–−6.59)	<.000001		
**Males**	1999	2012	−2.14* (−6.31–−0.09)	.049190	0.24 (−0.34–0.76)	.306739
	2012	2018	1.66 (−1.97–5.71)	.427914		
	2018	2021	16.10* (10.70–20.29)	.000400		
	2021	2023	−9.83* (−16.18–−2.27)	.013597		
**Race/Ethnicity**						
**Hispanics**	1999	2017	−2.12* (−3.14–−1.23)	<.000001	−0.59 (−1.29–0.09)	.094381
	2017	2021	15.59* (10.69–24.71)	.000400		
	2021	2023	−15.40* (−23.44–−5.18)	.001200		
**NH American Indians/Alaska Natives**	2007	2023	5.62* (2.38–10.27)	.002400	5.62* (2.38–10.27)	.002400
**NH Asians/Pacific Islanders**	1999	2018	−2.53* (−4.18–−0.66)	.040392	−0.73 (−1.7–0.43)	.14197
	2018	2021	20.58 (−5.28–27.07)	.101580		
	2021	2023	−11.84 (−23.38–6.89)	.131574		
**NH Blacks/African Americans**	1999	2014	−3.99* (−5.37–−2.95)	<.000001	−0.82* (−1.4–−0.22)†	.005599
	2014	2023	4.69* (2.58–7.94)	<.000001		
**NH Whites**	1999	2012	−2.44* (−3.91–−1.90)	.005599	−0.09 (−0.47–0.20)	.522695
	2012	2018	1.06 (−1.34–4.29)	.346331		
	2018	2021	15.83* (11.61–19.01)	<.000001		
	2021	2023	−9.74* (−13.93–−5.39)	<.000001		
**U.S. Census region**						
**Northeast**	1999	2015	−2.82* (−3.77–−2.12)	<.000001	−0.60* (−1.1–0.11)	.016397
	2015	2023	3.97* (1.91–7.10)	.000400		
**Midwest**	1999	2002	2.52 (−1.61–8.96)	.267147	−0.35 (−0.78–0.1)	.110778
	2002	2011	−4.06* (−8.06–−3.23)	.003999		
	2011	2018	0.41 (−1.54–2.46)	.662268		
	2018	2021	14.36* (9.93–17.57)	<.000001		
	2021	2023	−10.26* (−14.48–−5.31)	<.000001		
**South**	1999	2011	−2.77* (−5.37–−2.01)	.005599	0.14 (−0.33–0.48)	.445511
	2011	2018	0.76 (−1.48–4.29)	.435913		
	2018	2021	19.61* (14.96–23.06)	<.000001		
	2021	2023	−10.42* (−15.48–−5.66)	<.000001		
**West**	1999	2012	−1.80 (−7.63–6.98)	.100780	0.45 (−0.27–1.28)	.186363
	2012	2018	2.33 (−3.13–6.30)	.393521		
	2018	2021	16.63* (9.81–21.76)	.000800		
	2021	2023	−12.06* (−19.18–−3.45)	.006799		
**Urbanization**						
**Urban**	1999	2011	−2.81* (−6.17–−1.95)	.029994	−0.05 (−0.60–0.34)	.736253
	2011	2018	0.28 (−2.08–3.48)	.824635		
	2018	2020	16.88* (7.94–22.30)	<.000001		
**Rural**	1999	2003	2.36 (−0.35–8.83)	.089582	1.46* (0.98–2.01)	<.000001
	2003	2010	−3.83* (−8.33–−2.46)	.003199		
	2010	2018	0.97 (−0.61–3.47)	.182364		
	2018	2020	22.55* (14.83–27.58)	<.000001		

APC = annual percentage change, AAPC = average annual percentage change, 95% CI = 95% confidence interval, NH = non-Hispanic.

* Indicates APC significantly different from zero at α = 0.05. Urbanization data included from 1999–2020.

† For strata with a single Joinpoint segment, the APC equals the AAPC.

### 3.2. Sex-stratified mortality trends due to gastrointestinal hemorrhage and diabetes

Across all years, men had higher mortality (total AAMR 1.84) than women (1.15). Female mortality fell over time (AAPC −0.60*; 95% CI: −0.97–−0.23), whereas that among males remained unchanged (AAPC 0.24; 95% CI: −0.34–0.76). Specifically, AAMRs for women declined from 1.38 in 2002 to 0.92 in 2009 (APC −4.96*; 95% CI: −8.31–−3.79), were stable through 2018, then rose to 1.48 in 2021 (APC 16.25*; 95% CI: 12.71–18.81) before declining to 1.20 in 2023 (APC −10.25*; 95% CI: −14.06–−6.59). Male mortality rates dropped from 1.90 in 1999 to 1.52 in 2012 (APC −2.14*; 95% CI: −6.31–−0.09), remained stable until 2018, climbed to 2.62 by 2021 (APC 16.10*; 95% CI: 10.70–20.29), and then declined to 2.14 by 2023 (APC −9.83*; 95% CI: −16.18–−2.27). The trends in male and female mortality were not parallel (*P* < .05) (Fig. 1, Table 1, Table 2, Table S1, Supplemental Digital Content, https://links.lww.com/MD/R640, Table S10, Supplemental Digital Content, https://links.lww.com/MD/R640).

### 3.3. Mortality trends due to gastrointestinal hemorrhage and diabetes stratified by race and ethnicity

**Figure 2.**
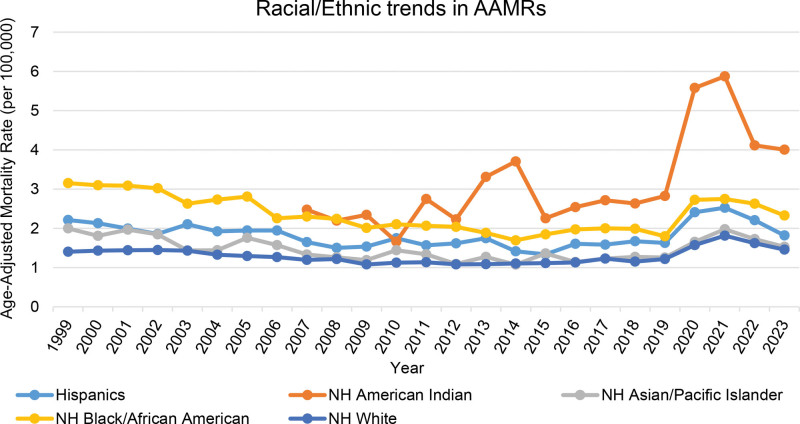
Racial and ethnic disparities in mortality from coexisting diabetes and gastrointestinal bleeding, 1999–2023; Age-adjusted mortality rates (AAMRs) per 100,000 U.S. adults aged ≥ 25 yr, stratified by race/ethnicity. NH American Indian mortality trends before 2007 were inaccessible due to data limitations. AAMR = age-adjusted mortality rate, NH = non-Hispanic.

Among racial/ethnic groups, non-Hispanic American Indian/Alaskan Natives had the highest overall AAMR (2.97) and experienced a notable increase post-2007 (AAPC 5.62*; 95% CI: 2.38–10.27). Non-Hispanic Black/African Americans had the second highest AAMRs (2.32), with a modest decline over time (AAPC −0.82; 95% CI: −1.41–−0.22). Hispanic AAMRs fell from 2.21 in 1999 to 1.58 in 2017 (APC −2.12*; 95% CI: −3.14–−1.23), surged to 2.53 in 2021 (APC 15.59*; 95% CI: 10.69–24.71), and dropped to 1.82 by 2023 (APC −15.40*; 95% CI: −23.44–−5.18). Asian/Pacific Islanders experienced a decline from 2.00 in 1999 to 1.27 in 2018 (APC −2.53*; 95% CI: −4.18–−0.66), followed by a non-significant rise in 2021 and a subsequent decline by 2023. Non-Hispanic Whites had the lowest rates (1.30) and showed similar late-period increases; after stability from 2012 to 2018, AAMR rose from 1.15 in 2018 to 1.81 in 2021 (APC 15.83*; 95% CI: 11.61–19.01) and then declined to 1.46 by 2023 (APC −9.74*; 95% CI: −13.93–−5.39). Black/African American populations followed mortality patterns distinct from those of White and Hispanic groups (*P* < .05), while other racial comparisons showed no significant differences (*P* > .05). (Fig. 2, Table 1, Table 2, Table S2, Supplemental Digital Content, https://links.lww.com/MD/R640, Table S10, Supplemental Digital Content, https://links.lww.com/MD/R64).

### 3.4. Urban versus rural mortality trends due to gastrointestinal hemorrhage and diabetes

**Figure 3.**
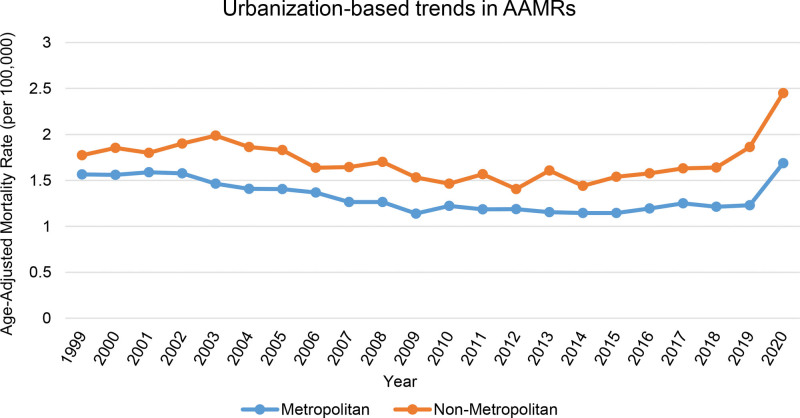
Urban–rural disparities in mortality from coexisting diabetes and gastrointestinal bleeding, 1999–2020; Age-adjusted mortality rates (AAMRs) per 100,000 U.S. adults aged ≥ 25 yr, stratified by metropolitan status. Urbanization-wise data is limited by CDC WONDER, and could not be attained beyond the year 2020. AAMR = age-adjusted mortality rate.

Mortality was consistently higher in rural (non-metropolitan) areas (total AAMR 1.72) than in urban (metropolitan) areas (1.31). Rural rates declined from 1.99 in 2003 to 1.46 in 2010 (APC −3.83*; 95% CI:−8.33–−2.46), then rose insignificantly until 2018. A significant uptick followed between 2018 (1.64) and 2020 (2.45) (APC 22.55*; 95% CI: 14.83–27.58). Urban AAMRs declined from 1.57 in 1999 to 1.18 in 2011 (APC −2.81*; 95% CI: −6.17–−1.95), stabilized through 2018, and rose sharply to 1.69 by 2020 (APC 16.88*; 95% CI: 7.94–22.30). Mortality trends in metropolitan and non-metropolitan areas were not parallel (*P* < .05) (Fig. 3, Table 1, Table 2, Table S3, Supplemental Digital Content, https://links.lww.com/MD/R640, Table S10, Supplemental Digital Content, https://links.lww.com/MD/R640).

### 3.5. Mortality trends due to gastrointestinal hemorrhage and diabetes stratified by U.S. census regions

**Figure 4.**
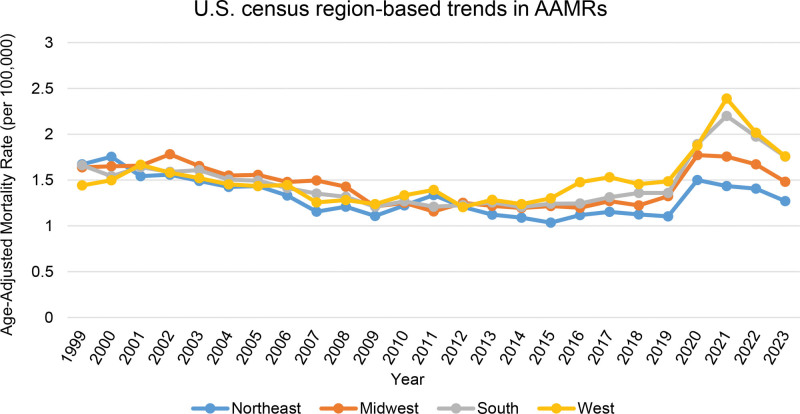
Regional disparities in mortality from coexisting diabetes and gastrointestinal bleeding, 1999–2023; Age-adjusted mortality rates (AAMRs) per 100,000 U.S. adults aged ≥ 25 yr, stratified by U.S. Census regions. AAMR = age-adjusted mortality rate.

The West region had the highest overall AAMR (1.53), followed by the South (1.48), the Midwest (1.43), and the Northeast (1.30) regions. Across the entire period, only the Northeast showed a significant decline (AAPC −0.60*; 95% CI: −1.10–−0.11). In the Northeast, AAMR fell from 1.67 in 1999 to 1.04 in 2015 (APC −2.82*; 95% CI: −3.77–−2.12) and then rose to 1.27 by 2023 (APC 3.97*; 95% CI: 1.91–7.10). The Midwest saw a decline from 1.78 in 2002 to 1.16 in 2011 (APC −4.06*; 95% CI: −8.06–−3.23), stability through 2018, a surge to 1.76 in 2021 (APC 14.36*; 95% CI: 9.93–17.57), and a subsequent decline to 1.48 in 2023 (APC −10.26*; 95% CI: −14.48–−5.31). The South exhibited similar patterns: a decline to 1.21 by 2011, stable rates through 2018, a rise to 2.20 in 2021 (APC 19.61*; 95% CI: 14.96–23.06), and a decline to 1.76 in 2023 (APC −10.42*; 95% CI: −15.48–−5.66). The West experienced modest changes in mortality rates until 2018, followed by an increase to 2.39 in 2021 (APC 16.63*; 95% CI: 9.81–21.76) and a decline to 1.76 in 2023 (APC −12.06*; 95% CI: −19.18–−3.45). All 4 census regions followed significantly different mortality trajectories over time (*P* < .05) (Fig. 4, Table 1, Table 2, Table S4, Supplemental Digital Content, https://links.lww.com/MD/R640, Table S10, Supplemental Digital Content, https://links.lww.com/MD/R640).

### 3.6. U.S. States with the highest and lowest AAMRs due to gastrointestinal hemorrhage and diabetes

**Figure 5.**
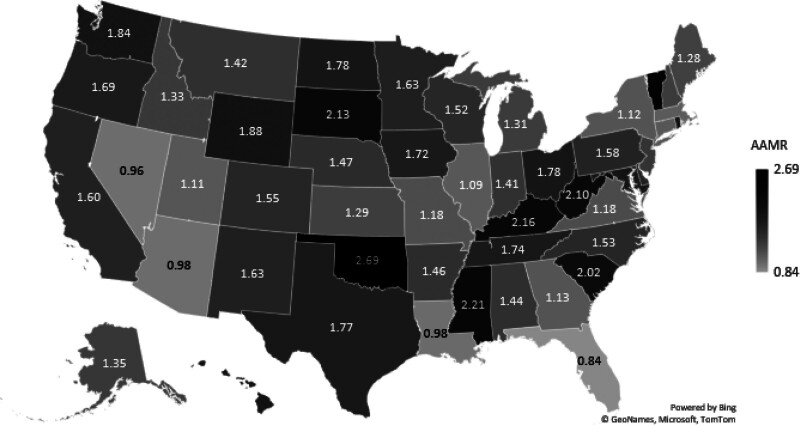
U.S. State-level disparities in mortality from coexisting diabetes and gastrointestinal bleeding, 1999–2023; total Age-adjusted mortality rates (AAMRs) per 100,000 U.S. adults aged ≥ 25 yr, stratified by U.S. Census regions. AAMR = age-adjusted mortality rate.

Among U.S. states, Oklahoma had the highest overall AAMR (2.69), followed by the District of Columbia (2.39), Vermont (2.36), Mississippi (2.21), and Kentucky (2.16). On the other hand, Florida (0.84), Nevada (0.96), Arizona (0.98), Louisiana (0.98), and Massachusetts (1.07) had the lowest AAMRs (Fig. 5, Table S5, Supplemental Digital Content, https://links.lww.com/MD/R640).

### 3.7. Overall mortality trend, where gastrointestinal hemorrhage was the underlying cause of death, with and without coexisting diabetes

When gastrointestinal hemorrhage was considered the underlying cause of death and diabetes mellitus as a contributory cause, similar to our main results, the overall AAMRs did not demonstrate a significant change between 1999 and 2023 (AAPC −0.06; 95% CI: −2.21–2.13). In contrast, when solely gastrointestinal hemorrhage-related AAMRs were analyzed, a significant decline was observed during the same period (AAPC −1.03*; 95% CI: −1.56–−0.52). Mortality trends based on gastrointestinal hemorrhage as the underlying cause with diabetes as a contributory cause were parallel to gastrointestinal hemorrhage-related mortality patterns without fatal diabetes (*P* > .05), but both differed from trends when gastrointestinal hemorrhage and diabetes were coexistent as either underlying or contributory causes of death (*P* < .05) (Tables S6–S9, Supplemental Digital Content, https://links.lww.com/MD/R640, Table S10, Supplemental Digital Content, https://links.lww.com/MD/R640).

## 4. Discussion

While mortality rates due to gastrointestinal hemorrhage alone have exhibited a net decline between 1999 and 2023, rates due to coexistent gastrointestinal hemorrhage and diabetes have not changed significantly. Overall, males and non-Hispanic American Indians/Alaskan Natives had the highest mortality rates. The West region and rural populations had the highest total AAMRs; states in the top 90th percentile (Oklahoma, District of Columbia, Vermont, Mississippi, and Kentucky) exhibited more than twice the AAMRs of those in the lower 10th percentile. Although some subgroup-specific changes were modest in magnitude, even small annual percentage changes can translate into substantial population-level mortality differences over extended follow-up periods.

Between 1999 and 2012, the decline in mortality rates may reflect advances in cardiovascular risk prevention, endoscopic techniques, and the management of gastrointestinal conditions during this period.^[19–21]^ Conversely, the spike in mortality from 2018 to 2021 may be attributed to the disruption in healthcare services during the COVID-19 pandemic.^[22]^ In addition, individuals with diabetes faced a markedly higher risk of severe outcomes from SARS-CoV-2 infection, driven by a combination of impaired immune defenses, virus-induced β-cell dysfunction, and the inflammatory cytokine storm.^[23]^ To make matters worse, endoscopic interventions, which can be life-saving in gastrointestinal hemorrhage, were also delayed over this time.^[24]^ The lack of improvement in gastrointestinal hemorrhage and diabetes mellitus-related AAMRs, in contrast to AAMRs from gastrointestinal hemorrhage alone, may be due to the influence of diabetes-related mortality in our analysis. However, this trend may also point to a possible gap in how recent progress in gastrointestinal bleeding outcomes has reached different patient groups, warranting further investigation into diabetes-specific factors that may limit mortality reduction.

Over the study period, male mortality rates were higher than those of females; mortality trends among both sexes were distinct. While the mortality rates declined among females, they remained unchanged among males. This pattern is interesting, as women often present with a greater prevalence of specific risk factors for diabetes and gastrointestinal hemorrhage.^[25,26]^ Nonetheless, a U.S. National Inpatient Sample database study highlighted that men with gastrointestinal bleeding, particularly esophageal variceal bleeding, are more likely to die than their female counterparts.^[27]^ Males also present with more advanced liver diseases, experience higher rates of alcohol use and hepatitis co-infection, and have lower estrogen-mediated hepatic protection.^[27]^ Furthermore, males tend to engage in health-seeking behaviors less frequently than females, often delaying or avoiding healthcare visits,^[28]^ leading to poorer health outcomes, including lower life expectancy and higher mortality rates. However, the extent to which these factors influence sex-specific mortality rates needs further evaluation.

With respect to races and ethnic origins, the highest AAMRs were observed among NH American Indian/Alaskan Native populations, followed by NH Black/African American adults. The unreliability of NH American Indians/Alaskan Natives data before 2007 reflects the historic limitations in data reporting that might have obscured the actual burden of diseases among this population.^[29]^ Nonetheless, the AAMRs for NH American Indians rose substantially following 2007. This may be attributed to the high incidence of diabetes, alcohol consumption, smoking, and socioeconomic disparities among these populations.^[29–31]^ Additionally, limited access to specialty care, geographic isolation, discriminatory distribution of resources, and chronic underfunding of the Indian Health Service can also explain the high mortality burden among NH American Indians.^[32–36]^ Similarly, high AAMRs among Blacks or African Americans mirror historically limited healthcare insurance coverage, lower access to preventive care, suboptimal diabetes control, and underutilization of therapies like SGLT2 inhibitors or endoscopic surveillance among them.^[34–36]^ However, due to a lack of granular data, we were unable to delineate the exact mechanisms driving racial and ethnic disparities related to gastrointestinal hemorrhage-associated mortality among patients with diabetes.

Rural regions consistently exhibited higher age-adjusted mortality rates due to concurrent diabetes and gastrointestinal bleeding; relevant trends were significantly different from those in urban areas. Similar to our findings, a 2022 study utilizing CDC WONDER data revealed that, as a contributory cause, diabetes-related mortality decreased by 13.5% in urban counties, while it increased by up to 8.9% in rural counties.^[37]^ This urban-rural disparity can be attributed to several factors. Firstly, people who live in rural areas face significant barriers in getting timely specialty care, such as gastroenterology and endocrinology services.^[38,39]^ The lack of health facilities and a smaller percentage of preventive care visits, with overall greater rates of poverty and unemployment, further impedes access to healthcare services in rural settings.^[40]^ According to previous literature, patients visiting rural hospitals with gastrointestinal hemorrhage are less likely to undergo lifesaving treatment procedures, such as endoscopy and colonoscopy, in comparison with urban hospitals.^[41]^ Addressing this rural-urban gap will require further investigation into relevant confounders. However, investment in specialty services beyond metropolitan centers is necessary to ensure that rural healthcare facilities across the U.S. are well-equipped to provide lifesaving procedures such as endoscopy.

In our regionally stratified analysis, every region displayed a distinct pattern of mortality. Nonetheless, we found that the overall mortality rates were the highest in the West and South regions. Generally, these regions also report elevated prevalence of cirrhosis and alcohol-associated liver disease, both of which are known to increase the risk of gastrointestinal bleeding.^[42]^ Additionally, many Southern U.S. states are frequently characterized as “medical deserts,” areas with lower median incomes and extensive healthcare shortages, which may significantly impair timely diagnosis and management of gastrointestinal complications in people with diabetes.^[43]^ It is also essential to understand that many of the high-mortality states, such as Oklahoma and Mississippi, had also resisted Medicaid expansion under the Affordable Care Act.^[44]^ On the other hand, the Northeastern states, such as Massachusetts, benefited from earlier Medicaid expansion and possess better healthcare infrastructure, contributing to their relatively lower overall mortality rates.^[44]^ Further population-level evidences are required to understand the role of region-specific risk prevalence, healthcare access, and insurance rate in driving regional disparities. However, to reduce the burden of mortality across the U.S. in general, and specifically among high-burden regions and states, region-specific public health measures need to be implemented.

Future researchers should aim to identify the factors contributing to the disproportional burden of gastrointestinal hemorrhage among patients with diabetes, with a particular focus on biological differences, healthcare access, structural injustices, and variations in treatment patterns across the United States. While we were unable to establish any direct causal relationship between diabetes and gastrointestinal hemorrhage, our findings support the idea that coexistent diabetes and gastrointestinal hemorrhage may impose higher risks of death than their individual occurrences. Nonetheless, the unchanged burden of mortality demands strategic public health measures, with a clear focus on vulnerable patient cohorts.

## 5. Limitations

It is important to acknowledge certain limitations of the CDC WONDER analyses while interpreting the findings of our paper. Firstly, this analysis was based on death certificate data, which is typically completed by individual physicians or coroners. The accuracy of comorbidity coding depends on the consistency and precision of its reporting, which cannot be independently verified. It is also essential to consider that the CDC WONDER database lacks information regarding clinical context, medications, comorbidities, and disease severity. The potential impact of unidentified comorbidities, which may have been underestimated, cannot be ruled out. Additionally, changes in coding practices over time may impact the accuracy of our analysis. Furthermore, the accuracy of the geographic data may have been influenced by out-of-state deaths and regional variations in death certification practices. Finally, we were unable to incorporate socioeconomic factors into our analysis, and this limits our ability to provide a robust account of their influence on observed trends.

## 6. Conclusion

In conclusion, while combined mortality rates from gastrointestinal hemorrhage and diabetes did not change between 1999 and 2023, they still contribute to a significant burden of mortality. The sociodemographic and geographical variations in mortality rates may reflect broader disparities in access to and utilization of gastrointestinal services. In addition to recognizing and addressing the mortality risks imposed by the coexistence of diabetes and gastrointestinal hemorrhage, strengthening healthcare infrastructure and ensuring equitable access to prevention, diagnosis, and care are imperative. Future research should clarify the pathways linking social and structural determinants of health to the variances in pertinent mortality patterns among the U.S. population.

## Author contributions

**Conceptualization:** Moiz Ul Haq Hashmi, Muddassir Khalid.

**Data curation:** Ramisha Chaudhary, Muhammad Riyyan, F.N.U. Farukhuddin.

**Formal analysis:** Ramisha Chaudhary, Sayed Jawad Hussaini, Ghulam Mustafa Ali Malik, Laiba Akhlaq.

**Investigation:** Krish Patel.

**Methodology:** Ramish Hannat, Muhammad Saeed Qazi.

## Supplementary Material






