# Supplementary material for: Trends and disparities in gastrointestinal hemorrhage-related mortality in individuals with diabetes in the United States from 1999 to 2023: A cross-sectional study
Source: Medicine (Baltimore). 2026 Apr 17;105(16):e48293. doi: 10.1097/MD.0000000000048293 (PMC13095341; doi:10.1097/MD.0000000000048293)
Supplement: Supplementary file 1 [file medi-105-e48293-s001.docx]

**Additional Information:**

**ICD-10 Codes**

| ***Diabetes Mellitus:*** |
| --- |
|  |
| E10-E14 (Diabetes mellitus) |
|  |
| ***Gastrointestinal Hemorrhage:*** |
|  |
| K92.0 (Hematemesis) |
| K92.1 (Melaena) |
| K92.2 (Gastrointestinal hemorrhage, unspecified) |
| K22.6 (Gastro-esophageal laceration-hemorrhage syndrome) |
| K25.0 (Gastric ulcer, acute with hemorrhage) |
| K25.2 (Gastric ulcer, acute with both hemorrhage and perforation) |
| K25.4 (Gastric ulcer, chronic or unspecified with hemorrhage) |
| K25.6 (Gastric ulcer, chronic or unspecified with both hemorrhage and perforation) |
| K26.0 (Duodenal ulcer, acute with hemorrhage) |
| K26.2 (Duodenal ulcer, acute with both hemorrhage and perforation) |
| K26.4 (Duodenal ulcer, chronic or unspecified with hemorrhage) |
| K26.6 (Duodenal ulcer, chronic or unspecified with both hemorrhage and perforation) |
| K27.0 (Peptic ulcer, site unspecified, acute with hemorrhage) |
| K27.2 (Peptic ulcer, site unspecified, acute with both hemorrhage and perforation) |
| K27.4 (Peptic ulcer, site unspecified, chronic or unspecified with hemorrhage) |
| K27.6 (Peptic ulcer, site unspecified, chronic or unspecified with both hemorrhage and perforation) |
| K28.0 (Gastrojejunal ulcer, acute with hemorrhage) |
| K28.2 (Gastrojejunal ulcer, acute with both hemorrhage and perforation) |
| K28.4 (Gastrojejunal ulcer, chronic or unspecified with hemorrhage) |
| K28.6 (Gastrojejunal ulcer, chronic or unspecified with both hemorrhage and perforation) |
| K29.0 (Acute hemorrhagic gastritis) |
| K62.5 (Hemorrhage of anus and rectum) |
