# Supplementary material for: Trends and disparities in gastrointestinal hemorrhage-related mortality in individuals with diabetes in the United States from 1999 to 2023: A cross-sectional study
Source: Medicine (Baltimore). 2026 Apr 17;105(16):e48293. doi: 10.1097/MD.0000000000048293 (PMC13095341; doi:10.1097/MD.0000000000048293)
Supplement: Supplementary file 2 [file medi-105-e48293-s002.pdf]

Table S1. Overall and sex-stratified annual age-adjusted mortality rates due to coexistent gastrointestinal hemorrhage and diabetes mellitus, United States, 1999-2023.

Age-Adjusted Rate (95% CI)			
Year	Overall	Female	Male
1999	1.60 (1.54–1.66)	1.41 (1.33–1.48)	1.90 (1.79–2.00)
2000	1.62 (1.56–1.67)	1.38 (1.31–1.45)	1.97 (1.86–2.07)
2001	1.63 (1.57–1.69)	1.45 (1.38–1.52)	1.91 (1.80–2.01)
2002	1.64 (1.58–1.70)	1.38 (1.31–1.45)	1.98 (1.87–2.08)
2003	1.55 (1.50–1.61)	1.35 (1.28–1.42)	1.89 (1.79–1.99)
2004	1.48 (1.42–1.53)	1.28 (1.21–1.34)	1.82 (1.73–1.92)
2005	1.49 (1.43–1.54)	1.22 (1.16–1.28)	1.84 (1.75–1.94)
2006	1.41 (1.35–1.46)	1.18 (1.11–1.24)	1.77 (1.68–1.86)
2007	1.33 (1.28–1.38)	1.11 (1.05–1.17)	1.61 (1.52–1.69)
2008	1.34 (1.29–1.39)	1.08 (1.02–1.14)	1.67 (1.58–1.76)
2009	1.22 (1.17–1.26)	0.92 (0.87–0.98)	1.53 (1.44–1.61)
2010	1.26 (1.22–1.31)	1.01 (0.96–1.07)	1.63 (1.54–1.71)
2011	1.24 (1.20–1.29)	1.04 (0.98–1.09)	1.56 (1.48–1.64)
2012	1.21 (1.17–1.26)	0.98 (0.93–1.03)	1.52 (1.44–1.60)
2013	1.22 (1.18–1.27)	0.94 (0.89–1.00)	1.58 (1.50–1.66)
2014	1.19 (1.14–1.23)	0.91 (0.86–0.96)	1.50 (1.43–1.58)
2015	1.19 (1.15–1.24)	0.97 (0.92–1.02)	1.57 (1.49–1.65)
2016	1.26 (1.22–1.31)	0.98 (0.93–1.03)	1.63 (1.55–1.71)
2017	1.33 (1.28–1.37)	1.01 (0.96–1.06)	1.73 (1.65–1.81)
2018	1.29 (1.24–1.33)	0.96 (0.91–1.01)	1.75 (1.67–1.83)
2019	1.33 (1.29–1.38)	1.05 (1.00–1.10)	1.68 (1.61–1.76)
2020	1.81 (1.76–1.86)	1.34 (1.28–1.40)	2.38 (2.29–2.47)
2021	2.01 (1.95–2.06)	1.48 (1.42–1.55)	2.62 (2.53–2.72)
2022	1.82 (1.76–1.87)	1.37 (1.31–1.43)	2.36 (2.27–2.45)
2023	1.63 (1.58–1.68)	1.20 (1.15–1.26)	2.14 (2.05–2.22)
Total	1.45 (1.44–1.46)	1.15 (1.14–1.16)	1.84 (1.82–1.85)

Table S2. Race/ethnicity-based annual age-adjusted mortality rates due to coexistent gastrointestinal hemorrhage and diabetes mellitus, United States, 1999-2023.

	Age-Adjusted Rate (95% CI)				
Year	Hispanic	NH American Indian	NH Asian	NH Black	NH White
1999	2.21 (1.88–2.54)	Unreliable	2.00 (1.56–2.52)	3.15 (2.86–3.44)	1.40 (1.34–1.46)
2000	2.13 (1.81–2.45)	3.34 (2.12–5.01)	1.81 (1.40–2.29)	3.10 (2.81–3.38)	1.43 (1.37–1.49)
2001	1.99 (1.70–2.29)	Unreliable	1.96 (1.56–2.44)	3.09 (2.80–3.37)	1.44 (1.38–1.50)
2002	1.86 (1.59–2.13)	2.85 (1.76–4.36)	1.85 (1.47–2.29)	3.02 (2.74–3.30)	1.44 (1.39–1.50)
2003	2.10 (1.82–2.39)	Unreliable	1.44 (1.11–1.83)	2.63 (2.37–2.89)	1.43 (1.37–1.49)
2004	1.93 (1.66–2.19)	2.42 (1.46–3.78)	1.44 (1.12–1.82)	2.73 (2.47–2.99)	1.32 (1.27–1.38)
2005	1.94 (1.69–2.20)	2.79 (1.78–4.14)	1.76 (1.42–2.16)	2.81 (2.55–3.07)	1.29 (1.24–1.35)
2006	1.95 (1.70–2.20)	Unreliable	1.58 (1.27–1.94)	2.26 (2.03–2.48)	1.27 (1.21–1.32)
2007	1.65 (1.42–1.88)	2.48 (1.57–3.71)	1.33 (1.05–1.66)	2.30 (2.07–2.53)	1.20 (1.14–1.25)
2008	1.50 (1.29–1.71)	2.20 (1.41–3.27)	1.26 (0.99–1.57)	2.24 (2.01–2.46)	1.22 (1.16–1.27)
2009	1.53 (1.33–1.74)	2.34 (1.53–3.44)	1.19 (0.94–1.48)	2.01 (1.80–2.22)	1.08 (1.03–1.13)
2010	1.75 (1.53–1.96)	1.66 (1.01–2.56)	1.44 (1.16–1.72)	2.10 (1.89–2.32)	1.13 (1.08–1.18)
2011	1.57 (1.37–1.76)	2.75 (1.84–3.95)	1.34 (1.08–1.60)	2.06 (1.85–2.27)	1.13 (1.08–1.19)
2012	1.61 (1.42–1.81)	2.23 (1.44–3.29)	1.09 (0.87–1.35)	2.04 (1.84–2.24)	1.08 (1.03–1.13)
2013	1.75 (1.55–1.95)	3.31 (2.38–4.49)	1.27 (1.04–1.51)	1.89 (1.70–2.08)	1.09 (1.04–1.14)
2014	1.41 (1.24–1.58)	3.70 (2.72–4.92)	1.07 (0.86–1.29)	1.69 (1.51–1.87)	1.10 (1.05–1.15)
2015	1.34 (1.18–1.50)	2.26 (1.53–3.20)	1.36 (1.13–1.59)	1.85 (1.67–2.03)	1.11 (1.06–1.16)
2016	1.60 (1.43–1.78)	2.54 (1.82–3.44)	1.14 (0.94–1.35)	1.97 (1.78–2.16)	1.13 (1.08–1.18)
2017	1.58 (1.42–1.75)	2.71 (1.95–3.68)	1.23 (1.02–1.43)	2.00 (1.81–2.18)	1.23 (1.18–1.28)
2018	1.67 (1.50–1.84)	2.63 (1.90–3.54)	1.27 (1.07–1.47)	1.99 (1.81–2.17)	1.15 (1.10–1.20)
2019	1.63 (1.47–1.79)	2.83 (2.07–3.77)	1.26 (1.06–1.45)	1.80 (1.63–1.97)	1.22 (1.17–1.27)
2020	2.41 (2.22–2.60)	5.58 (4.49–6.86)	1.66 (1.44–1.87)	2.73 (2.52–2.93)	1.57 (1.52–1.63)
2021	2.53 (2.33–2.72)	5.87 (4.71–7.24)	1.98 (1.74–2.22)	2.75 (2.54–2.96)	1.81 (1.75–1.88)

**2022**	2.21 (2.03–2.39)	4.11 (3.18–5.23)	1.73 (1.51–1.95)	2.63 (2.43–2.83)	1.62 (1.57–1.68)
**2023**	1.82 (1.66–1.98)	4.00 (3.10–5.09)	1.53 (1.33–1.73)	2.33 (2.14–2.51)	1.46 (1.41–1.51)
Total	1.83 (1.79–1.87)	2.97 (2.78–3.16)	1.45 (1.40–1.50)	2.32 (2.28–2.36)	1.30 (1.28–1.31)

Table S3. Urbanization-based annual age-adjusted mortality rates due to coexistent gastrointestinal hemorrhage and diabetes mellitus, United States, 1999-2020.

Age-Adjusted Rate (95% CI)		
Year	Metropolitan	Nonmetropolitan
1999	1.57 (1.50–1.63)	1.77 (1.63–1.92)
2000	1.56 (1.50–1.62)	1.85 (1.71–2.00)
2001	1.59 (1.52–1.65)	1.80 (1.66–1.94)
2002	1.58 (1.51–1.64)	1.90 (1.75–2.05)
2003	1.46 (1.40–1.52)	1.99 (1.84–2.14)
2004	1.41 (1.35–1.47)	1.86 (1.72–2.01)
2005	1.41 (1.35–1.46)	1.83 (1.69–1.97)
2006	1.37 (1.31–1.43)	1.64 (1.50–1.77)
2007	1.26 (1.21–1.32)	1.64 (1.51–1.78)
2008	1.27 (1.21–1.32)	1.70 (1.57–1.84)
2009	1.14 (1.09–1.19)	1.53 (1.41–1.66)
2010	1.22 (1.17–1.28)	1.46 (1.34–1.59)
2011	1.18 (1.13–1.24)	1.57 (1.44–1.69)
2012	1.19 (1.14–1.24)	1.41 (1.29–1.53)
2013	1.15 (1.11–1.20)	1.61 (1.48–1.73)
2014	1.15 (1.10–1.19)	1.44 (1.32–1.56)
2015	1.15 (1.10–1.19)	1.54 (1.42–1.66)
2016	1.20 (1.15–1.24)	1.58 (1.45–1.70)
2017	1.25 (1.20–1.30)	1.63 (1.51–1.76)
2018	1.21 (1.17–1.26)	1.64 (1.52–1.76)
2019	1.23 (1.18–1.28)	1.86 (1.73–2.00)
2020	1.69 (1.63–1.74)	2.45 (2.30–2.60)

**Total**	1.31 (1.30–1.32)	1.72 (1.69–1.75)

Table S4. U.S. Census region–based annual age-adjusted mortality rates due to coexistent gastrointestinal hemorrhage and diabetes mellitus, United States, 1999-2023.

	Age-Adjusted Rate (95%CI)			
YEAR	Northeast	Midwest	South	West
1999	1.67 (1.54–1.80)	1.64 (1.52–1.76)	1.66 (1.56–1.76)	1.44 (1.32–1.57)
2000	1.75 (1.62–1.89)	1.65 (1.53–1.77)	1.54 (1.45–1.64)	1.50 (1.37–1.62)
2001	1.54 (1.42–1.67)	1.66 (1.54–1.78)	1.64 (1.54–1.74)	1.67 (1.53–1.80)
2002	1.56 (1.43–1.68)	1.78 (1.66–1.91)	1.59 (1.49–1.69)	1.58 (1.45–1.71)
2003	1.49 (1.37–1.61)	1.65 (1.53–1.77)	1.61 (1.51–1.70)	1.52 (1.40–1.65)
2004	1.42 (1.31–1.54)	1.55 (1.43–1.67)	1.50 (1.41–1.60)	1.45 (1.33–1.57)
2005	1.44 (1.32–1.55)	1.56 (1.44–1.67)	1.49 (1.40–1.58)	1.43 (1.32–1.55)
2006	1.33 (1.22–1.44)	1.48 (1.37–1.59)	1.41 (1.32–1.50)	1.45 (1.33–1.56)
2007	1.16 (1.05–1.26)	1.50 (1.38–1.61)	1.35 (1.27–1.44)	1.26 (1.15–1.36)
2008	1.21 (1.10–1.32)	1.43 (1.32–1.54)	1.32 (1.24–1.40)	1.28 (1.18–1.39)
2009	1.11 (1.01–1.21)	1.21 (1.11–1.31)	1.21 (1.13–1.29)	1.24 (1.14–1.34)
2010	1.22 (1.12–1.33)	1.25 (1.15–1.35)	1.27 (1.19–1.35)	1.33 (1.23–1.44)
2011	1.34 (1.22–1.45)	1.16 (1.06–1.25)	1.21 (1.13–1.29)	1.39 (1.28–1.50)
2012	1.21 (1.10–1.31)	1.25 (1.15–1.35)	1.23 (1.15–1.30)	1.21 (1.11–1.30)
2013	1.12 (1.02–1.22)	1.22 (1.12–1.32)	1.26 (1.18–1.33)	1.29 (1.19–1.39)
2014	1.09 (0.99–1.19)	1.20 (1.10–1.29)	1.21 (1.13–1.28)	1.24 (1.14–1.33)
2015	1.04 (0.94–1.13)	1.22 (1.12–1.31)	1.24 (1.17–1.32)	1.30 (1.20–1.40)
2016	1.12 (1.02–1.22)	1.20 (1.10–1.29)	1.24 (1.17–1.32)	1.48 (1.37–1.58)
2017	1.15 (1.05–1.25)	1.27 (1.18–1.37)	1.31 (1.24–1.39)	1.53 (1.43–1.63)
2018	1.13 (1.03–1.22)	1.22 (1.13–1.32)	1.36 (1.29–1.44)	1.46 (1.36–1.56)
2019	1.10 (1.01–1.20)	1.33 (1.23–1.42)	1.36 (1.28–1.43)	1.49 (1.39–1.58)
2020	1.50 (1.39–1.61)	1.77 (1.66–1.88)	1.89 (1.81–1.98)	1.88 (1.77–1.99)
2021	1.43 (1.33–1.54)	1.76 (1.65–1.87)	2.20 (2.11–2.30)	2.39 (2.26–2.52)
2022	1.41 (1.30–1.51)	1.67 (1.56–1.78)	1.97 (1.89–2.06)	2.02 (1.90–2.13)
2023	1.27 (1.17–1.37)	1.48 (1.38–1.58)	1.76 (1.67–1.84)	1.76 (1.65–1.86)
Total	1.30 (1.28–1.32)	1.43 (1.41–1.46)	1.48 (1.47–1.50)	1.53 (1.50–1.55)

Table S5. U.S. state–wise total age-adjusted mortality rates due to coexistent gastrointestinal hemorrhage and diabetes mellitus, United States, 1999-2023.

State	Age-Adjusted Rate (95% CI)
Oklahoma	2.69 (2.56–2.81)
District of Columbia	2.39 (2.08–2.70)
Vermont	2.36 (2.09–2.62)
Mississippi	2.21 (2.08–2.34)
Kentucky	2.16 (2.06–2.26)
South Dakota	2.13 (1.91–2.35)
West Virginia	2.10 (1.95–2.24)
South Carolina	2.02 (1.93–2.12)
Wyoming	1.88 (1.60–2.15)
Hawaii	1.86 (1.70–2.02)
Rhode Island	1.86 (1.68–2.04)
Washington	1.84 (1.76–1.92)
Maryland	1.80 (1.71–1.88)
North Dakota	1.78 (1.56–2.00)
Ohio	1.78 (1.72–1.83)
Texas	1.77 (1.73–1.81)
Tennessee	1.74 (1.67–1.82)
Iowa	1.72 (1.62–1.82)
Oregon	1.69 (1.60–1.79)
Minnesota	1.63 (1.55–1.71)
New Mexico	1.63 (1.50–1.76)
California	1.60 (1.57–1.64)
Delaware	1.59 (1.40–1.78)
Pennsylvania	1.58 (1.53–1.62)
Colorado	1.55 (1.46–1.64)
North Carolina	1.53 (1.47–1.59)
Wisconsin	1.52 (1.44–1.59)
Nebraska	1.47 (1.34–1.59)
Arkansas	1.46 (1.36–1.57)
Alabama	1.44 (1.36–1.52)
Montana	1.42 (1.25–1.59)
Indiana	1.41 (1.34–1.47)
New Jersey	1.38 (1.32–1.43)
Alaska	1.35 (1.09–1.62)
Idaho	1.33 (1.20–1.47)

Michigan	1.31 (1.26–1.36)
New Hampshire	1.30 (1.16–1.45)
Kansas	1.29 (1.19–1.39)
Maine	1.28 (1.15–1.41)
Virginia	1.18 (1.12–1.24)
Missouri	1.18 (1.11–1.24)
Georgia	1.13 (1.07–1.18)
New York	1.12 (1.09–1.15)
Utah	1.11 (1.00–1.22)
Connecticut	1.10 (1.02–1.17)
Illinois	1.09 (1.05–1.13)
Massachusetts	1.07 (1.02–1.13)
Louisiana	0.98 (0.91–1.05)
Arizona	0.98 (0.92–1.03)
Nevada	0.96 (0.87–1.05)
Florida	0.84 (0.81–0.86)

Table S6. Annual deaths, population, and age-adjusted mortality rates when gastrointestinal hemorrhage was the underlying cause of death and diabetes mellitus was a contributory cause, United States, 1999-2023.

Year	Deaths	Population	Age-Adjusted Rate
1999	573	1.8E+08	0.31
2000	602	1.82E+08	0.33
2001	648	1.84E+08	0.35
2002	620	1.86E+08	0.34
2003	626	1.88E+08	0.34
2004	623	1.9E+08	0.33
2005	603	1.93E+08	0.31
2006	620	1.95E+08	0.31
2007	606	1.97E+08	0.3
2008	626	2E+08	0.29
2009	552	2.02E+08	0.27
2010	589	2.04E+08	0.28
2011	613	2.07E+08	0.28
2012	621	2.09E+08	0.28
2013	656	2.11E+08	0.28
2014	616	2.14E+08	0.24
2015	661	2.17E+08	0.27
2016	687	2.19E+08	0.28
2017	693	2.21E+08	0.28
2018	704	2.23E+08	0.27
2019	741	2.25E+08	0.27
2020	924	2.27E+08	0.34
2021	979	2.24E+08	0.36
2022	1059	2.25E+08	0.38
2023	904	2.27E+08	0.32

Table S7. Annual percentage changes (APCs) and average annual percentage changes (AAPCs) in age-adjusted mortality rates, where gastrointestinal hemorrhage was the underlying cause of death and diabetes mellitus was a contributory cause, United States, 1999-2023.

Annual Percentage Change							
Segment	Lower Endpoint	Upper Endpoint	APC	Lower CI	Upper CI	Test Statistic (t)	Prob > |t|
1	1999	2018	-1.4582*	-1.9241	-0.9902	-6.5405	0.000005
2	2018	2021	13.2488	-3.4805	32.8778	1.6422	0.118911
3	2021	2023	-5.3109	-17.5227	8.7090	-0.8339	0.415926
Average Annual Percentage Change							
Range	Lower Endpoint	Upper Endpoint	AAPC	Lower CI	Upper CI	Test Statistic~	P-Value~
Full Range	1999	2023	-0.0624	-2.2078	2.1300	-0.0564	0.955008

Table S8. Overall annual age-adjusted mortality rates solely due to gastrointestinal hemorrhage, United States, 1999–2023.

Year	Age-Adjusted Rate (95% CI)
1999	20.45 (20.24–20.66)
2000	19.98 (19.77–20.19)
2001	19.52 (19.32–19.72)
2002	18.89 (18.70–19.09)
2003	18.31 (18.12–18.51)
2004	17.32 (17.13–17.51)
2005	16.81 (16.63–16.99)
2006	16.02 (15.85–16.20)
2007	15.14 (14.97–15.31)
2008	14.77 (14.60–14.93)
2009	14.20 (14.04–14.36)
2010	14.45 (14.29–14.61)
2011	14.51 (14.35–14.67)
2012	14.23 (14.07–14.38)
2013	14.49 (14.33–14.65)
2014	14.36 (14.21–14.52)
2015	14.61 (14.45–14.76)
2016	14.81 (14.66–14.97)
2017	14.95 (14.80–15.11)
2018	15.03 (14.87–15.18)
2019	14.89 (14.74–15.04)
2020	17.36 (17.20–17.52)
2021	19.54 (19.36–19.71)
2022	17.81 (17.64–17.97)
2023	16.17 (16.01–16.32)

Table S9. Annual percentage changes (APCs) and average annual percentage changes (AAPCs) in gastrointestinal hemorrhage–related age-adjusted mortality rates, United States, 1999-2023.

Variable	Lower Endpoint	Upper Endpoint	APC (95% CI)	P-Value for APC	AAPC (95% CI)	P-Value for AAPC
Overall						
	1999	2009	-3.71*(-4.01 to -3.42)	< 0.000001	-1.03* (-1.56 to -0.52)	0.000091
	2009	2018	0.27 (-0.16 to 0.70)	0.203458		
	2018	2021	9.33*(5.44 to 13.36)	0.000115		
	2021	2023	-7.88*(-10.97 to -4.68)	0.000146		

Supplementary Table S10: Tests of parallelism were conducted using the built-in pairwise comparison options in the Joinpoint regression software. The table shows the results from the parallelism test comparing the relevant cohorts in our analysis.

Abbreviations: DM = diabetes mellitus, GI = gastrointestinal.

**Comparison**	**Numerator degrees of freedom**	**Denominator degrees of freedom**	**Number of permutations**	**p-value**	**Significance level (α)**	**Interpretation**
"Overall vs. Female"	"9"	"30"	"4500"	"0.001333"	"0.05"	"Trends are not parallel; mortality trajectories differ significantly."
"Overall vs. Male"	"7"	"34"	"4500"	"0.000222"	"0.05"	"Trends are not parallel; mortality trajectories differ significantly."
"Female vs. Male"	"9"	"30"	"4500"	"0.000222"	"0.05"	"Trends are not parallel; mortality trajectories differ significantly."
"Hispanics vs. Asian or Pacific Islander"	"5"	"38"	"4500"	"0.381778"	"0.05"	"Trends are parallel; no significant difference."
"Hispanics vs. Black or African American"	"7"	"34"	"4500"	"0.000444"	"0.05"	"Trends are not parallel; mortality trajectories differ significantly."
"Hispanics vs. White"	"9"	"30"	"4500"	"0.335556"	"0.05"	"Trends are parallel; no significant difference."
"Asian or Pacific Islander vs. Black or African American"	"7"	"34"	"4500"	"0.484667"	"0.05"	"Trends are parallel; no significant difference."
"Asian or Pacific Islander vs. White"	"9"	"30"	"4500"	"0.108222"	"0.05"	"Trends are parallel; no significant difference."
"Black or African American vs. White"	"7"	"34"	"4500"	"0.000444"	"0.05"	"Trends are not parallel; mortality trajectories differ significantly."
"Metro vs. Non-Metro"	"7"	"28"	"4500"	"0.022"	"0.05"	"Trends are not parallel; mortality trajectories differ significantly."
"Census Region 1: Northeast vs. Census Region 2: Midwest"	"9"	"30"	"4500"	"0.001556"	"0.05"	"Trends are not parallel; mortality trajectories differ significantly."
"Census Region 1: Northeast vs. Census Region 3: South"	"7"	"34"	"4500"	"0.000222"	"0.05"	"Trends are not parallel; mortality trajectories differ significantly."
"Census Region 1: Northeast vs. Census Region 4: West"	"7"	"34"	"4500"	"0.000444"	"0.05"	"Trends are not parallel; mortality trajectories differ significantly."

Comparison	Numerator degrees of freedom	Denominator degrees of freedom	Number of permutations	p-value	Significance level (α)	Interpretation
"Census Region 2: Midwest vs. Census Region 3: South"	"9"	"30"	"4500"	"0.000667"	"0.05"	"Trends are not parallel; mortality trajectories differ significantly."
"Census Region 2: Midwest vs. Census Region 4: West"	"9"	"30"	"4500"	"0.002444"	"0.05"	"Trends are not parallel; mortality trajectories differ significantly."
"Census Region 3: South vs. Census Region 4: West"	"7"	"34"	"4500"	"0.001778"	"0.05"	"Trends are not parallel; mortality trajectories differ significantly."
Overall, when GI was hemorrhage (underlying) + DM was a contributory cause of death<br><br>Versus<br><br>Overall, when both GI hemorrhage and diabetes were the underlying/contributory causes of death	“4”	“30”	“4500”	“0.013556”	“0.05”	"Trends are not parallel; mortality trajectories differ significantly."
Overall, when GI was hemorrhage (underlying) + DM was a contributory cause of death<br><br>Versus<br><br>Overall, when GI hemorrhage alone was the contributory cause, without diabetes	“7”	“34”	“4500”	“0.165111”	“0.05”	"Trends are parallel; no significant difference."
Overall, when both GI hemorrhage and diabetes were the underlying/contributory causes of death<br><br>Versus<br><br>Overall, when GI hemorrhage alone was the contributory cause, without diabetes	“7”	“34”	“4500”	“0.000222”	“0.05”	"Trends are not parallel; mortality trajectories differ significantly."
